# Gene Editing in *Ganoderma lucidum*: Development, Challenges, and Future Prospects

**DOI:** 10.3390/jof11040310

**Published:** 2025-04-14

**Authors:** Shiqi He, Yuanchao Liu, Zhi Zhang, Manjun Cai, Yufan Hao, Huiping Hu

**Affiliations:** 1 National Health Commission Science and Technology Innovation Platform for Nutrition and Safety of Microbial Food, Guangdong Provincial Key Laboratory of Microbial Safety and Health, State Key Laboratory of Applied Microbiology Southern China, Institute of Microbiology, Guangdong Academy of Sciences, Guangzhou 510070, China; sk928020275sk@163.com (S.H.); liuyc1020@163.com (Y.L.); zhangzhi@gdim.cn (Z.Z.); caimanjun@gdim.cn (M.C.); asdf19845714278@foxmail.com (Y.H.); 2 Guangdong Yuewei Biotechnology Co., Ltd., Shaoguan 512029, China

**Keywords:** gene editing, *Ganoderma lucidum*, CRISPR/Cas9, edible fungi

## Abstract

As an emerging and innovative technology, gene-editing technology has been widely applied in crop breeding, human disease treatment, animal model research, drug and vaccine development, and microbial engineering. We mainly introduce the development of gene-editing technology, the application of clustered regularly interspaced short palindromic repeat/Cas9 (CRISPR/Cas9) in *Ganoderma lucidum* breeding, the current challenges and optimization strategies in the use of gene-editing technology in *Ganoderma* breeding, as well as the current status of gene-editing technology in *Ganoderma* breeding. Finally, the future research directions and innovative strategies that gene editing may explore in *Ganoderma* breeding are prospects given the existing background, future research directions, and innovative strategies that gene editing may explore in *Ganoderma* breeding prospects.

## 1. Introduction

*Ganoderma lucidum*, a valuable medicinal macro fungus, produces bioactive compounds, including polysaccharides, ganoderic acids (GA), and triterpenoids, with demonstrated immunomodulatory, antitumor, and antioxidant properties [1,2]. These bioactive metabolites have established its importance in pharmaceutical applications and health maintenance. Current research prioritizes functional gene identification and metabolic engineering to enhance bioactive compound production.

Traditional breeding approaches (artificial domestication, mutagenesis, and hybridization) have been employed for strain improvement but are limited by: (i) prolonged breeding cycles, (ii) unpredictable mutagenesis outcomes, (iii) inefficient mutant screening, and (iv) technical complexity [2,3]. These constraints necessitate the development of precise breeding technologies to generate strains with improved yield, stability, and stress resistance.

Gene-editing technologies have revolutionized *G. lucidum* breeding by enabling precise genetic modifications and significantly accelerating strain development compared to conventional methods. This review systematically examines: (i) the evolution of gene editing platforms, (ii) optimization strategies for fungal applications, and (iii) current implementations and persistent challenges in *G. lucidum* genetic improvement programs. The integration of these advanced tools has transformed the pace and precision of medicinal mushroom breeding.

## 2. The Development of Gene-Editing Technology

Gene-editing technology primarily refers to the application of genetic engineering techniques that enable precise modifications to an organism’s genome. This technology facilitates the insertion, deletion, replacement, or modification of DNA, thereby altering specific traits of the organism. In the practical application of *G. lucidum* breeding, gene-editing technology is predominantly utilized to achieve gene silencing of particular genes for research purposes.

### 2.1. First Generation: Zinc Finger Nucleases (ZFNs)

Initially developed in the mid-1990s, ZFNs represented the first programmable gene editing tool. Created by fusing zinc finger proteins with the cleavage domain of *Fok* I restriction endonuclease, ZFNs enabled targeted DNA double-strand breaks (DSBs) [4]. Despite their innovation, ZFNs were limited by design complexity, high production costs, and significant off-target effects, restricting their widespread application [5,6].

### 2.2. Second Generation: Transcription Activator-like Effector Nucleases (TALENs)

Emerging in 2011, TALENs offered improved design flexibility and an improved targeting range [7]. Composed of TALE domains fused with *Fok* I nuclease, TALENs demonstrated lower cytotoxicity compared to ZFNs while maintaining similar editing efficiency [8]. However, challenges persisted in the complexity of their construction, delivery efficiency, and production costs [9].

### 2.3. Third Generation: Clusters of Regularly Spaced Short Palindromic Repeats/Cas9 Protein Systems (CRISPR/Cas9 Systems)

The revolutionary CRISPR/Cas9 system, particularly popularized after 2012, transformed gene editing through its simplicity, versatility, and efficiency [10]. Utilizing RNA-guided DNA recognition, this system significantly reduced the complexity of targeting genetic sequences. CRISPR/Cas9 systems operate through DNA cleavage followed by repair through non-homologous end joining (NHEJ) or homology-directed repair (HDR), with the potential for precise genomic modifications [11,12].

### 2.4. Precision Gene Editing Tools

Recent advances have produced more precise editing technologies (see Table S1):

Base Editors (BEs): Developed in 2016–2017, cytosine and adenine base editors enable single nucleotide conversions (C-to-T or A-to-G) without requiring DSBs, substantially reducing indel formation [13,14]. Further optimizations have enhanced editing efficiency while maintaining high product purity [15,16,17,18].

Prime Editors (PEs): Introduced in 2019, prime editing combines Cas9 nickase with reverse transcriptase to enable all types of genetic modifications without DSBs or donor DNA templates [19]. Successive generations (PE1 to PE7) have achieved enhanced efficiency and reduced off-target effects through enzyme optimization and the incorporation of DNA repair modulators [20,21,22].

Now, gene-editing technologies have revolutionized multiple fields, including biomedical research (disease modeling, gene therapy, and drug discovery) [23,24,25,26], agriculture (crop improvement and livestock genetic enhancement) [27,28,29], and fungal research (functional genomics and metabolic engineering in species like *G. lucidum*) [30].

Despite remarkable progress, several challenges remain, such as delivery methods for in vivo applications, off-target effects, and editing precision. Research now focuses on enhancing delivery systems, improving specificity, expanding targeting scope, and addressing ethical implications. These advancements promise to further expand the applications of gene editing in biomedicine, agriculture, and biotechnology.

**Figure 1:**
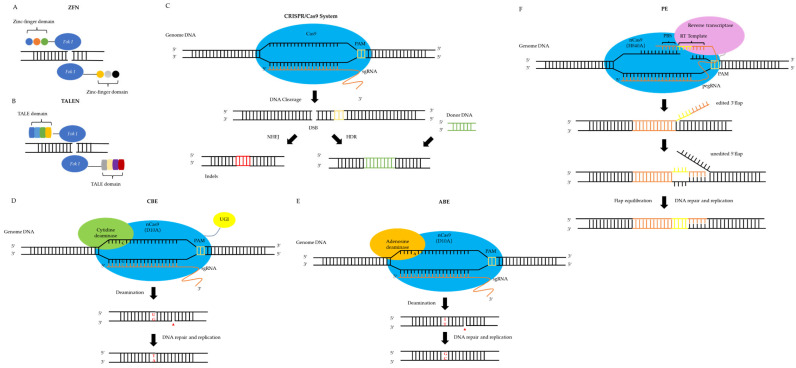
Working principle of gene-editing technologies. (**A**) ZFN: zinc finger nuclease; (**B**) TALEN: transcription activator-like effector nuclease; (**C**) CRISPR/Cas9: Clusters of Regularly Spaced Short Palindromic Repeats/Cas9 Protein Systems; NHEJ: non-homologous end joining (a type of DNA repair mechanism); HDR: homology-directed repair (a type of DNA repair mechanism); PAM: protospacer adjacent motif (used for Cas9 protein recognition of target DNA sequences); and sgRNA: single guide RNA (used to recruit Cas9 protein to the target site); (**D**) CBE: cytosine base editor; UGI: uracil glycosylase inhibitor; (**E**) ABE: adenine base editor; (**F**) PE: prime editor; PBS: primer binding site; RT Template: reverse transcript template; and pegRNA: prime editing guide RNA.

In this section, the developmental trajectory of genome editing technologies is briefly reviewed, encompassing ZFNs, TALENs, the CRISPR/Cas9 system, and precision editing platforms derived from CRISPR/Cas architectures. The operational mechanisms of these genome editing tools, along with the evolutionary progression of precision editing systems, are systematically summarized (see Figure 1 and Table 1).

## 3. Application of CRISPR/Cas9 System in *G. lucidum*

In 2017, the CRISPR/Cas9 system was first implemented in *G. lucidum* through protoplast transformation using a codon-optimized construct containing the endogenous *gpd* promoter, *Trichoderma reesei* terminator, and carboxin resistance marker [37]. The *ura3* gene, encoding orotidine-5′-phosphate decarboxylase, was targeted using *in vitro* transcribed sgRNA, enabling the selection of transformants via 5′-FOA resistance [38]. While this pioneering study demonstrated successful gene editing, the reliance on *in vitro* transcribed sgRNA introduced potential efficiency limitations due to nucleic acid degradation during synthesis and delivery. The development of continuous *in vivo* gRNA transcription systems has been pursued to overcome the limitations of *in vitro* approaches. Pol III promoters have been identified as potential candidates for sgRNA expression in *G. lucidum*, offering advantages in transcript stability and editing efficiency. However, the characteristic T-stretch termination signals of Pol III promoters generate heterogeneous transcripts with poly-U tails, which may impair CRISPR/Cas9 functionality. Consequently, the systematic evaluation of Pol III promoter variants is required to identify optimal configurations for efficient genome editing [39].

In 2019, through the comparative analysis of U6 promoter sequences across species, conserved regulatory elements were identified and employed to drive sgRNA expression in *G. lucidum* by Wang et al. [40]. The engineered system, incorporating HDV ribozyme for transcript processing, achieved 21.5% editing efficiency at the *ura3* locus, with improved performance relative to non-optimized controls. Successful application was demonstrated in ganoderic acid biosynthesis genes (*cyp5150l8*, *cyp505d13*), establishing the U6 promoter’s utility for fungal genome editing. The further exploration of regulatory elements is required to expand the genetic toolbox for *G. lucidum*.

In 2020, the functional characterization of calcineurin-responsive transcription factors (*glcrz1* and *glcrz2*) in *G. lucidum* was achieved through CRISPR/Cas9-mediated knockout [41]. While *glcrz1* disruption specifically impaired calcium-mediated GA biosynthesis regulation, *glcrz2* knockout significantly reduced both mycelial growth and GA production, revealing distinct metabolic roles for these paralogs. Concurrently, editing efficiency was enhanced through strategic intron incorporation in the Cas9 expression cassette, with the endogenous *gpd* intron increasing *ura3* targeting efficiency 10.6-fold [42]. The feasibility of multiplexed editing was demonstrated through dual sgRNA delivery, achieving 36.7% efficiency at the *ura3* locus. These studies established critical methodologies for transcriptional regulator analysis and editing optimization in *G. lucidum*.

In 2022, a CRISPR/Cas9-based in situ complementation system was developed to functionally characterize *gl26097*, a C2H2-type zinc finger transcription factor in the calmodulin-calcineurin pathway [43]. Knockout strains were generated and subsequently complemented via HDR-mediated reintegration, restoring GA production to wild-type levels. The targeted complementation of *cyp5150l8* (lanosterol to GA conversion) increased GA yields, confirming its biosynthetic role. While demonstrating precise genetic restoration, the system requires optimization to address off-target effects and low complementation efficiency.

In 2023, the implementation of ribonucleoprotein (RNP) complexes in *G. lucidum* genome editing was demonstrated to overcome the limitations of plasmid-based systems [44,45]. RNP delivery achieved 100% editing efficiency at the *ura3* locus, with superior performance relative to plasmid transformation (4–18 mutants/10^7^ protoplasts). While RNP efficacy showed sgRNA-dependent variability, membrane permeability enhancement through Triton X-100 treatment significantly improved editing outcomes [38]. Notably, plasmid degradation artifacts were observed to potentially impact fungal viability, underscoring the advantage of RNP approaches for clean genetic modification.

In 2024, efficient genome editing (about 7–8%) was achieved in *G. lucidum* through optimized protoplast transformation conditions, including 0.2 μM sorbitol buffer (pH 7.0) and a 10:1 protoplast-to-RNP ratio during *catA* gene targeting [46].

In 2025, a CRISPR/Cas9 RNP delivery system was employed to target mating-type genes (*A1/A2*) in *G. lucidum*, enabling cross-nuclear editing, as evidenced by 26 binucleate and 5 mononucleate transformants [47]. However, progressive resistance loss was observed during subculturing, indicating CRISPR-induced genomic instability and declining editing efficiency over time.

## 4. The Limitations of Gene-Editing Technology in Fungi at Present

### 4.1. Low Genetic Transformation Efficiency

The complex genetic background and low regeneration efficiency of *G. lucidum* present significant challenges for CRISPR/Cas system implementation, necessitating the optimization of transformation methodologies. Two primary approaches have been established: (i) *Agrobacterium tumefaciens*-mediated transformation (ATMT) enables efficient large-fragment integration but requires extensive labor, and (ii) protoplast-mediated transformation (PMT) facilitates multi-copy insertion yet suffers from low regeneration rates and stringent enzyme requirements [30,48].

Transformation efficiency is highly dependent on recipient materials, with mycelia demonstrating superior performance in *Agaricus bisporus* [49] and protoplasts demonstrating superior performance in *Hypsizygus marmoreus* [50] when using optimized AMAT conditions. Critical parameters affecting efficiency include: (i) *Agrobacterium*-protoplast ratios (optimal 1000:1) and co-culture conditions (26 °C with 0.3 mM acetosyringone) [50], and (b) membrane permeability enhancers (0.006% Triton X-100) and the incubation time of RNPs with stain [51]. Selection markers must be carefully chosen to avoid regeneration inhibition [52].

These findings underscore the necessity of species-specific protocol optimization, recipient material selection, and parameter standardization to maximize editing efficiency in fungal systems.

### 4.2. Low Efficiency of Gene Editing

The multicellular nature of edible fungi presents unique challenges for genome editing, particularly when targeting dikaryotic strains where simultaneous nuclear editing is required to achieve complete modification. Monokaryon isolation through spore suspension plating or protoplast regeneration remains technically demanding and resource-intensive. Transformation material selection critically impacts editing outcomes, necessitating optimized delivery strategies. Three principal delivery approaches have been established for fungal gene editing systems, each offering distinct advantages for overcoming these multicellular constraints.

Three principal delivery strategies have been developed for fungal genome editing applications. First, single-vector systems incorporating both CRISPR/Cas9 and sgRNA components enable efficient plasmid transformation, reducing delivery complexity while permitting multiplexed targeting through co-transformation [53]. Second, stable Cas9-expressing strains can be combined with *in vitro* transcribed sgRNAs, allowing flexible target switching through sgRNA replacement [38]. Third, RNP complexes offer transient activity through post-editing degradation, eliminating persistent foreign genetic material [44].

Several limitations persist across current delivery approaches. Plasmid-based systems require extensive promoter optimization and codon adaptation to ensure functional protein expression, with large vector sizes potentially compromising transformation efficiency. In addition, the requirements for selectable markers and the labor-intensive optimization process remain significant constraints for plasmid-based approaches [45]. Furthermore, plasmid degradation through endogenous nuclease activity may lead to genomic instability or reduced editing efficiency [45,54]. In *G. lucidum*, these challenges have been partially addressed through the use of endogenous regulatory elements, with the Pol III-type U6 promoter driving sgRNA expression and the *gpd* promoter controlling Cas9 transcription [53].

The transformation of *in vitro* transcribed sgRNA into Cas9-expressing cells presents multiple technical challenges, including costly sgRNA synthesis, complex assembly procedures, and susceptibility to nucleic acid degradation during delivery, all of which significantly impair editing efficiency [37,40]. In contrast, RNP delivery overcomes limitations associated with variable Cas9 expression and sgRNA transcription rates. The transient nature of RNPs prevents host persistence and minimizes off-target effects through post-editing degradation [55,56]. However, residual DNA contaminants in Cas9 protein preparations may cause unintended genomic insertions in *G. lucidum*, necessitating optimized purification protocols to ensure editing precision [45].

The molar ratio of RNPs critically influences transformation efficiency across fungal species. In *Pleurotus ostreatus*, RNP concentrations of 5 μg, 10 μg, and 20 μg yielded transformant efficiencies of 100%, 50%, and 27%, respectively [57]. Similarly, *Trichoderma reesei* required suitable concentration (100 and 170 nM) for detectable editing, achieving 100% efficiency at optimal concentrations [51]. In other research, dose-dependent effects were consistently observed, with maximal *ura3* editing in *G. lucidum* requiring 1000 ng Cas9 per 0.5 μg sgRNA [45]. Systematic optimization identified 220.6 and 294 nM as the ideal RNP range in *G. lucidum*, producing 35 transformants per 10^7^ protoplasts [44]. These findings demonstrate the necessity of species-specific RNP titration to balance delivery efficiency and resource utilization.

### 4.3. Low Precision of DNA Repair

The DNA repair landscape in filamentous fungi exhibits distinct characteristics, with NHEJ representing the dominant pathway despite HDR’s superior accuracy [58,59]. The genetic disruption of NHEJ mechanisms, while increasing HDR frequency, often results in growth defects and genomic instability [52]. In *G. lucidum*, RNP-induced DSBs are primarily repaired via NHEJ, generating variable indels and potential ectopic insertions from host- or vector-derived sequences [45]. Notably, NHEJ inhibition fails to sufficiently activate HDR components, maintaining low HDR: NHEJ ratios [60]. A comparative analysis of *G. lucidum* revealed that HDR efficiency (4.2 times higher than NHEJ)when offered donor DNA is constrained by donor DNA length (>2 kb reduces efficiency) [61]. The targeted knockout of *ku70* confirmed NHEJ predominance, with its silencing significantly enhancing HDR utilization [59]. These findings underscore the necessity for (i) optimized donor DNA design (<2 kb), (ii) NHEJ pathway modulation, and (iii) species-specific repair mechanism characterization to improve editing precision.

## 5. Optimization Strategies to Overcome the Limitations of Gene Editing in *G. lucidum*

CRISPR/Cas9 technology has emerged as a transformative genome editing platform, enabling precise DNA sequence modifications with unprecedented efficiency. Currently, the CRISPR/Cas9 system and its derivative editors have been widely applied in mammals [62,63,64], yeast [65,66,67], *Arabidopsis thaliana* [68,69,70], *Oryza sativa* [27,71,72,73], *Taraxacum mongolicum*, and *Rehmannia glutinosa* [74]. Compared to these species, applying this gene-editing technology in *G. lucidum* breeding still holds significant potential and room for development.

### 5.1. Gene Silencing

In the field of gene silencing, researchers have successfully utilized CRISPR/Cas9 technology to achieve gene knockout in the *G. lucidum* genome. Following the silencing of target genes, functional studies are conducted through phenotypic screening or by analyzing the expression levels of the target gene and related genes within the same pathway [37,40,42,43,75].

However, several technical constraints persist in CRISPR/Cas applications for *G. lucidum*, including limited targeting scope, suboptimal editing efficiency, off-target effects, and low protoplast regeneration rates. Current optimization strategies focus on: (i) expanding targetable sequences through Cas protein engineering, (ii) enhancing precision via codon optimization and nuclear localization signal incorporation, and (iii) improving fidelity using high-specificity Cas variants. These approaches aim to address the key challenges in fungal genome editing while maintaining editing efficacy.

#### 5.1.1. Lift the Restriction of PAM

The targeting scope of CRISPR systems has been substantially expanded through protein engineering approaches. The directed evolution of SpCas9 yielded variants with relaxed PAM requirements: (i) NG-PAM recognizing variants [76], (ii) SpG/SpRY with broadened recognition (NRN > NYN) [77], and (iii) specialized variants (VQR/VRQR for NGA-PAM; VRER for NGCG-PAM; and xCas9 for NGN-PAM) [78,79,80]. Additional Cas orthologs have been harnessed to further diversify targeting options, including: (a) SaCas9 (NNGRRT-PAM) and its KKH variant (NNNRRT-PAM) [81,82,83], (b) LbCas12a (TTTV-PAM) [84,85], (c) BthCas12b (ATTN-PAM) [86], and (d) AacCas12b (TTN-PAM) [86]. These engineered nucleases have collectively overcome PAM constraints while maintaining editing precision.

#### 5.1.2. Improve Gene Editing Efficiency

Editing efficiency in *G. lucidum* can be enhanced through codon optimization and nuclear localization signal (NLS) engineering. Species-specific codon adaptation is critical, as demonstrated by the failure of human-optimized Cas9 in *Trichoderma reesei* [87]. Optimal editing requires: (i) codon usage frequency matching to *G. lucidum*’s translational machinery and (ii) NLS optimization (copy number, type selection, and insertion site) to ensure proper nuclear localization, with reported ninefold efficiency improvements [15]. These strategies, validated in mammalian and plant systems, provide a framework for *G. lucidum* genome editing optimization.

#### 5.1.3. Reduce Off-Target Effects

Off-target effects in CRISPR/Cas systems represent a significant challenge: that unintended editing caused by sgRNA mismatches at non-target sequences and random editing mismatches at non-target sequences independent of sgRNA, which can induce genomic instability and unintended phenotypic consequences [88]. The development of high-fidelity Cas variants such as SpCas9-HF has substantially improved editing specificity through engineered reductions in non-target DNA binding affinity [79]. Optimal sgRNA design remains critical, with parameters including GC content below 35% to minimize mismatch potential [89] and computational screening using tools like Cas-Offinder [90], GUIDE-seq [91], and Digenome-seq [92] to eliminate sequences with predicted off-target activity. Delivery method selection further influences specificity, where RNP complexes offer advantages over plasmid-based systems through transient activity and avoidance of overexpression artifacts [93,94]. These combined approaches provide a comprehensive framework for minimizing off-target effects while maintaining editing efficiency in fungal systems.

### 5.2. Gene Overexpression

*G. lucidum* contains numerous beneficial metabolites, and one of its breeding objectives is to achieve efficient and stable production of these compounds. Therefore, the CRISPR/Cas9 system can silence inhibitory genes within metabolic synthesis pathways, activating the expression of upstream and downstream genes in a specific pathway. However, limited research has employed the CRISPR/Cas9 system to target and activate specific genes directly. Moreover, indirectly activating other genes through the CRISPR/Cas9 system may inadvertently affect gene expression in different pathways. Consequently, additional strategies can be explored to achieve targeted gene overexpression within shorter growth cycles, including constructing overexpression vectors for transformation, developing suitable chassis cells, and establishing CRISPR activation systems.

#### 5.2.1. Construct Overexpression Vectors to Improve Gene Expression Level

The first strategy involves constructing overexpression plasmids and achieving gene overexpression through PMT or AMT methods. The plasmid-based overexpression of *G. lucidum* endogenous genes, including *HMGR* (2-fold GA increase) [95], *D9desA* (1.2-fold GA enhancement) [96], and *LS* (6.1-fold GA elevation) [97], has demonstrated significant metabolic engineering potential. In addition, the *gpd* promoter has been particularly effective at driving gene expression, as evidenced by 75.73% increased triterpenoid production through *Gl-aact* and *CYP51* overexpression [98]. Chassis cell systems offer complementary advantages for recombinant protein production, combining genetic stability with rapid growth and cost efficiency [30]. *Escherichia coli* has been successfully employed for the high-yield expression of *G. lucidum* polysaccharide biosynthesis enzymes (PGM, UGPG), with culture conditions systematically optimized to maximize enzyme yield and characterize structural properties [99]. The heterologous expression of *Vitreoscilla* hemoglobin further enhanced oxygen-dependent bioprocesses in *G. lucidum*, enabling subsequent increases in extracellular polysaccharide production through the coordinated overexpression of glycosyltransferase genes [100].

#### 5.2.2. Develop CRISPR Activation System for Overexpression

CRISPR-based transcriptional activation systems have been developed by fusing catalytically inactive dCas9 with multiple activation domains, enabling the simultaneous upregulation of target genes without repeated vector construction [101]. The CRISPR-Act 2.0 system demonstrated enhanced activation (3–4-fold) through the MCP-MS2-mediated recruitment of additional VP64 copies to dCas9-VP64 complexes [102]. Further optimization led to CRISPR-Act 3.0, which combined dCas9-VP64 with 10xGCN4-SunTag and TAD modules, achieving 60-fold activation of the rice *OsTPR* gene and up to 140-fold activation of proanthocyanidin pathway genes when using the gRNA 2.0 scaffold [103]. These systems provide powerful tools for multiplexed gene activation in eukaryotic systems. They also provide a reference solution for the dilemma of *G. lucidum* breeding.

## 6. Conclusions and Perspectives

*G. lucidum* is valued in medicine for its bioactive compounds exhibiting anti-inflammatory, anticancer, antioxidant, and hypoglycemic properties [104]. Therapeutically relevant metabolites include (i) ethanol-extractable fermentation products (GFE) demonstrating hepatoprotective effects [105]; (ii) polysaccharides (GLP) with neuroprotective potential against neurodegenerative disorders [106]; and (iii) triterpenoids, glycoproteins, and unsaturated fatty acids [107]. Current metabolic engineering strategies employ either the heterologous expression of biosynthetic genes in microbial systems [99,108,109] or co-culture with *Lactobacillus plantarum* for enhanced production [110]. Advanced genome editing technologies are now being implemented to optimize strain development and metabolic output.

CRISPR/Cas9 is now widely used for functional genomics and metabolic engineering in *G. lucidum*, offering advantages over ZFNs/TALENs in design simplicity, cost, efficiency, and specificity. However, technical limitations remain for its application in *G. lucidum* breeding programs.

Monokaryotic strain isolation is typically performed prior to genetic transformation to ensure complete nuclear genome targeting, as dikaryotic strains may yield reduced editing efficiency due to partial genome modification. In *G. lucidum*, monokaryon screening remains technically challenging, commonly involving spore plating or protoplast isolation coupled with clamp connection analysis for verification. Critical optimization parameters include: (i) dikaryotic transition monitoring identification, (ii) microscopic observation protocol optimization, (iii) spore concentration standardization system construction, and (iv) protoplast regeneration efficiency enhancement. Additional considerations encompass species confirmation via ITS sequencing during spore-based methods and the maintenance of protoplast integrity during isolation procedures. The post-mitotic dilution effects must be accounted for when editing dikaryotic strains, while monokaryon isolation from established dikaryons presents significant technical hurdles.

Additionally, the mating-type genes of filamentous fungi can be analyzed to verify monokaryotic status in isolated strains. However, genetic transformation efficiency limitations often complicate positive transformant screening and reduce editing efficacy. Imprecise DNA repair mechanisms may introduce frameshift, missense, or synonymous mutations, potentially causing genomic instability. Off-target effects further compromise editing specificity through the unintended modification of non-target genes. Current optimization strategies include: (i) experimental condition refinement, (ii) codon optimization, (iii) nuclear localization signal incorporation, (iv) delivery system improvement, and (v) homology-directed repair enhancement to increase both editing efficiency and precision.

Base editing enables precise nucleotide conversions in *G. lucidum* without donor DNA or DSBs, exhibiting higher specificity than CRISPR/Cas9 [111,112,113]. This approach facilitates gene silencing via stop codon introduction [114] and expression modulation through ORF targeting [115]. Optimal sgRNA design using computational tools (CRISPRscan, CRISPR-BETS, and PE-Designer) is essential. In addition, fusion transposons also have the potential to become a new and efficient gene-editing method. For example, Tn7-like transposases can be guided to target sites by the CRISPR/Cas9 system to insert DNA fragments of up to 10 kb [116].

Artificial intelligence (AI) is increasingly being integrated with gene-editing technologies to enhance their precision and efficiency. Machine learning algorithms can analyze multi-omics data (proteomic, transcriptomic, and epigenetic) along with nucleic acid structural predictions to optimize several key aspects: (i) Cas protein-directed evolution, (ii) sgRNA sequence design, and (iii) effector complex engineering. Such AI-guided approaches enable the development of improved editing systems through the computational prediction of optimal protein-RNA configurations, significantly enhancing target recognition while minimizing off-target effects. Furthermore, the AI-assisted optimization of editing components has been shown to increase editing efficiency and product yield in later-stage applications.

In summary, this article provides a comprehensive overview of existing gene-editing technologies. It also elaborates on the applications of the CRISPR/Cas9 system in *G. lucidum*, the current challenges encountered, and the proposed optimization strategies to overcome these limitations. Finally, innovative ideas and unique perspectives for future research directions are presented based on the current research context, offering scientific evidence and theoretical support for designing future *G. lucidum* breeding strategies.

## Figures and Tables

**Table 1:** Overview of Gene-Editing Technology.

Gene-Editing Technology	Working Principle	Advantage	Disadvantage	Application
ZFNs	Zinc finger domains recognize and bind to specific DNA sequences, followed by dimerization of the *Fok* I endonuclease domain to execute cleavage activity.	Specific, targeting specific sequences for cleavage.	Constrained targeting range due to sequence recognition preferences; Complex design requirements for multi-finger arrays; cytotoxicity from excessive DNA damage response activation; and off-target effects from promiscuous heterodimer formation.	Human disease treatment and crop trait improvement, etc. [31,32,33].
TALENs	TALE domain specifically recognizes and binds to target DNA sequences, followed by dimerization of the *Fok* I nuclease domain to induce site-specific DNA cleavage at the predetermined genomic locus.	Simpler for design requirements and higher targeting specificity.	Complexity in design and construction; prohibitive production costs; low delivery efficiency of TALENs systems; and cytotoxic effects.	Disease modeling, plants, and livestock improvement, etc. [34,35,36].
CRISPR/Cas9	The target DNA sequence is recognized and bound by the Cas protein under the guidance of sgRNA, resulting in the induction of double-strand breaks at the designated genomic locus.	Higher specificity and simpler design.	High dependency on PAM sequences; off-target effects; and inability to achieve precise single-base editing.	Crop trait improvement, drug development, and disease treatment, etc. [23,24,25,26,27,28,29,30].

## Data Availability

No new data were created or analyzed in this study. Data sharing is not applicable to this article.

## Supplementary Materials

The following supporting information can be downloaded at: https://www.mdpi.com/article/10.3390/jof11040310/s1, Table S1: The development overview and optimization strategies of base editors and prime editors.
