# Supplementary material for: Gene Editing in Ganoderma lucidum: Development, Challenges, and Future Prospects
Source: J Fungi (Basel). 2025 Apr 14;11(4):310. doi: 10.3390/jof11040310 (PMC12029067; doi:10.3390/jof11040310)
Supplement: Supplementary file 1 [file jof-11-00310-s001.zip › jof-3548686-supplementary.pdf]

Supplementary information

Table S1. The development overview and optimization strategies of base editors and prime editors.

Gene editors		Optimization strategies	Optimization principles
CBE	BE1[1]	Fusion of rAPOBEC1 and dCas9	Achieve C-to-T substitution
	BE2[1]	Added UGI copies	Inhibit UNG and prevent the excision of U:G base pairs during DNA repair
	BE3[1]	Replaced dCas9 with nCas9	Increase the proportion of repair using the non-sgRNA-targeted strand as a template
	BE4[2]	Optimized the linker length and fused multiple copies of UGI	Inhibit UNG and optimize the protein folding process
	BE4max[3]	Codon optimization and NLS modification	Optimize protein expression and signal transduction processes
ABE	ABE7.10[4]	Fusion of TadA and nCas9	Achieve A-to-G substitution
	ABEmax[3]	Codon optimization and NLS modification	Optimize protein expression and signal transduction processes
	ABE8e[5]<br>ABE8s[6]	Directed evolution of Tad7.10	Enhance the deamination activity of deaminase
PE	PE1[7]	Fused reverse transcriptase, nCas9 and pegRNA	Genome editing through reverse transcription based on CRISPR/Cas9.
	PE2[7]	Directed evolution of M-MLV reverse transcriptase	Enhanced thermal stability of the enzyme, affinity for templates and RNA substrates, and processivity of the enzyme
	PE3[7]	Design additional sgRNAs to introduce nicks in the non-target strand	Promote the use of the target strand as a template for damage repair during DNA repair
	PE4[8]<br>PE5[8]	Fused MLH1dn to PE	Inhibition of the DNA mismatch repair mechanism

Gene editors	Optimization strategies	Optimization principles
PE4max[8]<br>PE5max[8]	Optimization of RT codons and linker length, Cas9 amino acid mutations, and addition of nuclear localization sequences	Enhance nuclease activity and optimize the signal transduction process
PE6[9]	Directed evolution of reverse transcriptase	Enhance the delivery efficiency of the prime editing system in host cells
PE7[10]	Fusion with the small RNA-binding exonuclease protection factor La protein	Enhance RNA stability

References

1. Komor, A.C.; Kim, Y.B.; Packer, M.S.; Zuris, J.A.; Liu, D.R. Programmable editing of a target base in genomic DNA without double-stranded DNA cleavage. *Nature* **2016**, *533*, 420-424, doi:10.1038/nature17946.
2. Komor, A.C.; Zhao, K.T.; Packer, M.S.; Gaudelli, N.M.; Waterbury, A.L.; Koblan, L.W.; Kim, Y.B.; Badran, A.H.; Liu, D.R. Improved base excision repair inhibition and bacteriophage Mu Gam protein yields C:G-to-T: A base editors with higher efficiency and product purity. *Sci Adv* **2017**, *3*, eaao4774, doi:10.1126/sciadv.aao4774.
3. Koblan, L.W.; Doman, J.L.; Wilson, C.; Levy, J.M.; Tay, T.; Newby, G.A.; Maianti, J.P.; Raguram, A.; Liu, D.R. Improving cytidine and adenine base editors by expression optimization and ancestral reconstruction. *Nature Biotechnology* **2018**, *36*, 843-846, doi:10.1038/nbt.4172.
4. Gaudelli, N.M.; Komor, A.C.; Rees, H.A.; Packer, M.S.; Badran, A.H.; Bryson, D.I.; Liu, D.R. Programmable base editing of A•T to G•C in genomic DNA without DNA cleavage. *Nature* **2017**, *551*, 464-471, doi:10.1038/nature24644.
5. Richter, M.F.; Zhao, K.T.; Eton, E.; Lapinaite, A.; Newby, G.A.; Thuronyi, B.W.; Wilson, C.; Koblan, L.W.; Zeng, J.; Bauer, D.E.; et al. Phage-assisted evolution of an adenine base editor with improved Cas domain compatibility and activity. *Nature Biotechnology* **2020**, *38*, 883-891, doi:10.1038/s41587-020-0453-z.
6. Gaudelli, N.M.; Lam, D.K.; Rees, H.A.; Solá-Esteves, N.M.; Barrera, L.A.; Born, D.A.; Edwards, A.; Gehrke, J.M.; Lee, S.-J.; Liquori, A.J.; et al. Directed evolution of adenine base editors with increased activity and therapeutic application. *Nature Biotechnology* **2020**, *38*, 892-900, doi:10.1038/s41587-020-0491-6.
7. Anzalone, A.V.; Randolph, P.B.; Davis, J.R.; Sousa, A.A.; Koblan, L.W.; Levy, J.M.; Chen, P.J.; Wilson, C.; Newby, G.A.; Raguram, A.; et al. Search-and-replace genome editing without double-strand breaks or donor DNA. *Nature* **2019**, *576*, 149-157, doi:10.1038/s41586-019-1711-4.
8. Chen, P.J.; Hussmann, J.A.; Yan, J.; Knipping, F.; Ravisankar, P.; Chen, P.-F.; Chen, C.; Nelson, J.W.; Newby, G.A.; Sahin, M.; et al. Enhanced prime editing systems by manipulating cellular determinants of editing outcomes. *Cell* **2021**, *184*, 5635-5652.e5629, doi:10.1016/j.cell.2021.09.018.
9. Doman, J.L.; Pandey, S.; Neugebauer, M.E.; An, M.; Davis, J.R.; Randolph, P.B.; McElroy, A.; Gao, X.D.; Raguram, A.; Richter, M.F.; et al. Phage-assisted evolution and protein engineering yield compact, efficient prime editors. *Cell* **2023**, *186*, 3983-4002.e3926, doi:10.1016/j.cell.2023.07.039.
10. Yan, J.; Oyler-Castrillo, P.; Ravisankar, P.; Ward, C.C.; Levesque, S.; Jing, Y.; Simpson, D.; Zhao, A.; Li, H.; Yan, W.; et al. Improving prime editing with an endogenous small RNA-binding protein. *Nature* **2024**, *628*, 639-647, doi:10.1038/s41586-024-07259-6.
